# Retracted: Inhibitory Effect of Ursolic Acid on Proliferation and Migration of Renal Carcinoma Cells and Its Mechanism

**DOI:** 10.1155/2022/9826832

**Published:** 2022-12-19

**Authors:** Computational Intelligence and Neuroscience

*Computational Intelligence and Neuroscience* has retracted the article titled “Inhibitory Effect of Ursolic Acid on Proliferation and Migration of Renal Carcinoma Cells and Its Mechanism” [1] due to concerns that the peer review process has been compromised.

Following an investigation conducted by the Hindawi Research Integrity team [2], significant concerns were identified with the peer reviewers assigned to this article; the investigation has concluded that the peer review process was compromised. We therefore can no longer trust the peer review process, and the article is being retracted with the agreement of the Chief Editor.
